# Sustained Exposure to *Helicobacter pylori* Lysate Inhibits Apoptosis and Autophagy of Gastric Epithelial Cells

**DOI:** 10.3389/fonc.2020.581364

**Published:** 2020-10-29

**Authors:** Yang He, Cunlong Wang, Xiulin Zhang, Xuancheng Lu, Jin Xing, Jianyi Lv, Meng Guo, Xueyun Huo, Xin Liu, Jing Lu, Xiaoyan Du, Changlong Li, Zhenwen Chen

**Affiliations:** ^1^ School of Basic Medical Sciences, Capital Medical University, Beijing Key Laboratory of Cancer Invasion & Metastasis Research, Beijing, China; ^2^ Laboratory Animal Center, Chinese Center for Disease Control and Prevention, Beijing, China; ^3^ Institute for Laboratory Animal Resources, National Institutes for Food and Drug Control, Beijing, China

**Keywords:** *Helicobacter pylori*, apoptosis, autophagy, gastric epithelial cell, carcinogenesis

## Abstract

*Helicobacter pylori* is designated as a class I carcinogen of human gastric cancer following long-term infection. During this process, *H. pylori* bacteria persist in proliferation and death, and release bacterial components that come into contact with gastric epithelial cells and regulate host cell function. However, the impact of long-term exposure to *H. pylori* lysate on the pathological changes of gastric cells is not clear. In this study, we aimed to investigate the regulation and mechanisms involved in gastric cell dysfunction following continuous exposure to *H. pylori* lysate. We co-cultured gastric cell lines GES-1 and MKN-45 with *H. pylori* lysate for 30 generations, and we found that sustained exposure to *H. pylori* lysate inhibited GES-1 cell invasion, migration, autophagy, and apoptosis, while it did not inhibit MKN-45 cell invasion or migration. Furthermore, Mongolian gerbils infected with *H. pylori* ATCC 43504 strains for 90 weeks confirmed the *in vitro* results. The clinical and *in vitro* data indicated that sustained exposure to *H. pylori* lysate inhibited cell apoptosis and autophagy through the *Nod1-NF-κB/MAPK-ERK/FOXO4* signaling pathway. In conclusion, sustained exposure to *H. pylori* lysate promoted proliferation of gastric epithelial cells and inhibited autophagy and apoptosis *via Nod1-NF-κB/MAPK-ERK/FOXO4* signaling pathway. In the process of *H. pylori-*induced gastric lesions, *H. pylori* lysate plays as an “accomplice” to carcinogenesis.

## Introduction

*Helicobacter pylori* (*H. pylori*) is designated as a class I carcinogen of human gastric cancer (1), and it can survive for prolonged periods in the acidic gastric environment. *H. pylori* infection is closely related to gastritis, peptic ulcer, gastric cancer, gastric mucosa-associated lymphoid tissue (MALT) lymphoma, and even some extragastric diseases (2–5). It is generally believed that the diseases induced by *H. pylori* infection are caused by living bacteria. *H. pylori* induces defective autophagy or inhibits autophagy to promote its own colonization (6, 7). Moreover, *H. pylori* is involved in migration, invasion, autophagy, and apoptosis, eventually leading to gastric cancer (8, 9). *H. pylori* promotes the malignant transformation of the host cells by transporting cytotoxin-associated gene product A (CagA), an oncoprotein, to cells through the type IV secretion system (T4SS) (10–12). Furthermore, *H. pylori* secretes vacuolating cytotoxin A (VacA) (13) and destroys the activity of lysosomal calcium channels in host cells, which leads to the formation of dysfunctional enlarged lysosomes and allows *H. pylori* to colonize in the stomach and, thus, escape from eradication therapy (14). In addition, the outer membrane vesicles (OMVs) released by *H. pylor*i contain a variety of bacterial toxins and antigens (15), which are absorbed by gastric epithelial cells (16) and enhance the carcinogenic potential of *H. pylori* (17).

During long-term infection by *H. pylori*, a large number of bacteria persist in proliferation and death. Massive bacterial compositions maintain contact with and stimulate gastric epithelial cells. These bacterial components enter the host cell in multiple ways, such as receptor recognition and OMVs, regulate cell survival and metabolism, and lead to pathological diseases of the gastric mucosal barrier (18–20). During bacterial disintegration in the stomach, various components act on endothelial cells simultaneously. It has been reported that *H. pylori* lysate promotes hepatocellular carcinoma (HSC) cell proliferation and liver fibrosis (21). Further, *Helicobacter suis* lysate regulates the apoptosis of gastric epithelial cells (22). To date, most reports have investigated the mechanisms of *H. pylori*-induced gastric diseases by infecting living bacteria or a single bacterial virulence factor to cell lines for 6 h to 72 h (8, 18, 21, 22). However, these cannot simulate the effects of long-term stimulation of *H. pylori* on gastric cells. Because *H. pylori* cannot survive co-cultures with cells for an extended time, long-term co-cultures with gastric epithelial cells using *H. pylori* lysate instead of living bacteria are used to simulate the regulatory effects of persistent infection on cells. In this process, the effects of *H. pylori* lysate are also important.

In this study, *H. pylori* lysate was prepared by ultrasonic lysis and was co-cultured with gastric epithelial cells for 30 consecutive generations to investigate the underlying mechanisms involved in its cellular regulatory activity *in vitro* and *in vivo*. Our data demonstrate that sustained exposure of gastric epithelial cells to *H. pylori* lysate promoted proliferation and inhibited autophagy and apoptosis, and it may further lead to malignant transformation in gastric epithelial cells.

## Materials and Methods

### Bacterial Culture and Preparation of Bacterial Lysate

The *H. pylori* strain American Type Culture Collection (ATCC) 43504 (cagA^+^, vacA^+^) was obtained from the National Institutes for Food and Drug Control, Beijing. *H. pylori* was grown on Colombian agar plates (OXOID, UK, CM0331B) containing 5% sterile and defibrated sheep blood (MRC, China, CCS30037.01) at 37°C under microaerophilic conditions for 48 h.

*H. pylori* was scraped off the plate and washed twice with phosphate buffer saline (PBS) (KeyGen BioTECH, China, KGB5001), then mixed with PBS, and ultrasonic lysis was performed. We used the bicinchoninic acid (BCA) method to detect protein concentration. The lysate was stored at -20°C until use.

### Cell Lines and Cell Culture

The human normal gastric epithelial cell line GES-1 and human gastric adenocarcinoma cell line MKN-45 were purchased from Beijing Dingguo Changsheng Biotechnology Co., Ltd. Cells were grown in DMEM (Corning, USA, 10-013-CVR) supplemented with 10% fetal bovine serum (FBS) (PAN, Germany, P30-3302) and 1% penicillin/streptomycin binary antibody solution (KeyGen BioTECH, China, KGY0023) in a humidified environment and under 5% CO_2_ at 37°C.

GES-1 cells and MKN-45 cells of the experimental group were cultured in medium added with *H. pylori* lysate for 30 consecutive generations. The other conditions were consistent with those of the control group. The untreated normal cells were labeled as B-GES-1 and B-MKN-45, which were cultured for 30 consecutive generations. The cells co-cultured with *H. pylori* lysate for 30 generations were labeled as Cul_30_-GES-1 and Cul_30_-MKN-45, respectively.

### Cell Treatment

A total of 4×10^5^ Cul_30_-GES-1 and B-GES-1, Cul_30_-MKN-45, and B-MKN-45 cells were seeded into 6-well plates. After the cells were attached, normal DMEM, DMEM containing *H. pylori* lysate, or DMEM containing *H. pylori* (6×10^6^ CFU/mL) (23) was separately added to the 6-well plates for a total of 2 mL per well, and cells were incubated for 24 h.

### Determining the Optimum Concentration of *H. pylori* Lysate to Be Co-Cultured With Cells

The optimum concentration of *H. pylori* lysate to be co-cultured with cells was determined by MTT. B-GES-1 or B-MKN-45 cells were digested with 0.25% trypsin and washed with PBS. The cell suspension concentration was adjusted to 2.5×10^4/^mL using DMEM medium containing 10% FBS. The cells were inoculated in 96-well plates with a volume of 100 μL per well. The edge wells of the 96-well plate were filled with 200 μL sterile PBS solution, and the culture was continued for 6 h to allow the cells to adhere. After the cells were attached, the medium was discarded, and the cells were washed twice with PBS solution. In the experimental group, medium containing different concentrations of *H. pylori* lysate was added (0.5 μg/mL, 1 μg/mL, 1.5 μg/mL, 2 μg/mL, 2.5 μg/mL, 3 μg/mL, 3.5 μg/mL, 4 μg/mL, 4.5 μg/mL, 5 μg/mL), in a volume of 200 μL per well, and the control group received 200 μL of normal medium containing an equal amount of PBS solution. Five sub-wells were set for each experimental group and control group. The cells were cultured for another 72 h. After 72 h of culture, the culture medium was discarded. Cells were washed twice with PBS solution. Sterile MTT solution (Solarbio, China, M1020) was added to the well in a final volume of 100 μL. The cells were cultured for 4 h. The solution in the 96-well plate was discarded and 100 μL dimethyl sulfoxide (DMSO) was added to each well. The plate was placed on a shaking table for 10 min to dissolve the methylamine precipitate. Absorbance of each well was read at 490 nm using a microplate reader of a spectrophotometer to measure the cell quantity.

### Cell Proliferation Assay

In this study, gastric cells were co-cultured with *H. pylori* or *H. pylori* lysate. The cell proliferation of Cul_30_-GES-1 and B-GES-1, Cul_30_-MKN-45, and B-MKN-45 were compared by two methods. First, the cells were plated into 96-well plates and then cultured in medium containing *H. pylori* lysate or *H. pylori* for 24 h or 48 h. Then MTT colorimetric assay was carried out. Second, plate cloning assay was used. Cul_30_-GES-1 and B-GES-1 (500 cells/well) were placed in 6-well plates and maintained in DMEM medium containing 10% FBS. After 14 days, the cells were fixed and stained by crystal violet. Visible colonies were then counted by Image J software. Each well was assessed in triplicate.

### Wound Healing Assay

B-GES-1 and Cul_30_-GES-1 were placed in 6-well plates and maintained in DMEM medium containing 10% FBS. The cell concentration was 5×10^5^ cells/mL and 2 mL volume was added per well. When cells reached 90% confluence, a wound was generated, the medium was discarded, and the cells were washed twice with PBS solution to remove any floating cells. In the experimental group, serum free medium containing *H. pylori* lysate or *H. pylori* was added at a volume of 2 mL per well, and the control group received 2 mL of serum free medium. The cells were incubated for 24 h. The gap distances were measured to assess the capacity of the cells to migrate.

### Transwell Cell Migration and Invasion Assay

Transwell migration chamber was used for the migration assay. After treatment with *H. pylori* lysate or *H. pylori*, Cul_30_-GES-1 and B-GES-1, Cul_30_-MKN-45, and B-MKN-45 cells (3×10^4^ cells in 200 μL) were placed in the upper chamber in serum-free medium. Medium containing 10% FBS was added to the lower chamber. The cells, following a 24-h incubation, were fixed and stained with 0.1% crystal violet and counted. The assays were performed in triplicate.

For the invasion assay, a total of 3×10^4^ cells were seeded into the upper chamber of a Transwell invasion chamber with serum-free media, while medium containing 10% FBS was added to the lower chamber. The cells, after a 24-h incubation, were fixed and stained with Hoechst (Solarbio, China, B8040) for 10 min in a dark environment. The number of cells that invaded from the upper chamber were counted using Image J software.

### Cytokine Level Analysis

Cul_30_-GES-1, B-GES-1 and Cul_30_-MKN-45, B-MKN-45 cells were treated with *H. pylori* lysate or *H. pylori* for 24 h. Cytokine level analysis was performed by Shanghai Universal Biotech Co., Ltd. The Luminex detection assays were used to measure the expression of each factor in the samples. According to previous reports, eight cytokines in Cul_30_-GES-1 and B-GES-1 cells related to autophagy, apoptosis, migration, or invasion were selected for detection, including TNF-α, CCL-20, CCL-28, CXCL-2, IFN-γ, TFPI, SLPI, and FAS. Three cytokines, CCL-20, CCL-28, and CXCL-2, in Cul_30_-MKN-45 and B-MKN-45 cells associated with migration and invasion were also detected.

### mCherry-EGFP-LC3 Transfection

Two milliliters of Cul_30_-GES-1 and B-GES-1 cell suspension with a density of 2.5 × 10^4^ cells/mL was prepared in complete medium and added to a 6-well plate. After the cells were attached, the old medium was discarded, the cells were washed with PBS, and medium containing a 50 μL titer of 10^8^ TU/mL mCherry-EGFP-LC3 lentivirus (purchased from SyngenTech Co., Ltd) and 8 μg/mL Polybrene was added to the culture. After 48 h of infection, the fluorescence expression of cells was observed by fluorescence microscopy. When the efficiency of cell infection reached about 80%, the cells were cultured in medium containing *H. pylori* lysate or *H. pylori* for an additional 24 h. The images of mCherry-EGFP-LC3 transfected cells were observed by laser scanning confocal microscopy. The autophagy flux was measured by the color change of mCherry-EGFP.

### Hoechst Staining

After treatment with *H. pylori* lysate or *H. pylori*, Cul_30_-GES-1 and B-GES-1 cells were washed with PBS three times. Next, cells were stained with Hoechst 33342 (10 μg/mL) for 10 min. Nuclear morphologic changes were examined under a fluorescence microscope.

### Apoptosis Assay

Flow cytometry was used to detect the effects of *H. pylori* lysate on the apoptosis of Cul_30_-GES-1 and B-GES-1 cells using the Annexin V-PE/7-AAD Apoptosis Detection Kit (Vazyme, China, A213-01) according to the manufacturer’s recommendations. The rate of apoptosis was analyzed using LSR Fortessa Flow Cytometer at 488 nm.

### Evaluation of Gene Expression *via* the TCGA Database

The gene mRNA expression data of 132 gastric adenocarcinoma cases (17 cases of *H. pylori* infection and 115 cases without *H. pylori* infection) were downloaded from the TCGA database (https://cancergenome.nih.gov/). Log_2_ transformation and Z-correction were performed to normalize the expression value of each gene.

### Reverse Transcriptase Polymerase Chain Reaction

A TRIzol Reagent (Vazyme, China, R401-01) was used to isolate total RNA from Cul_30_-GES-1 and B-GES-1 cells. Single-stranded DNA was prepared from 1 μg total RNA using reverse transcriptase-bound oligonucleotide (DT) primers. Each cDNA sample (2 μL) was subjected to reverse transcriptase polymerase chain reaction (RT-PCR) amplification using specific primers as detailed in **Supplementary Table 1**. The data were collected and analyzed. The values were compared with the experimental controls after being normalized to those of GAPDH.

### Western Blot

Following treatment with *H. pylori* lysate or *H. pylori*, Cul_30_-GES-1 and B-GES-1, Cul_30_-MKN-45, and B-MKN-45 cells were lysed by Radio Immunoprecipitation Assay (RIPA) Lysis Buffer (Solarbio, China, R0010) on ice for 30 min. The cell lysate was centrifuged at 13400×g at 4°C for 15 min. The supernatants were collected, and the protein concentration was measured with a BCA protein kit (Thermo Scientific, USA, A53225). The lysate was mixed with PBS and 5× SDS loading buffer (ROBY, China, RBU114-2) and heated at 99°C for 10 min. Western blots were performed on 8% or 10% SDS-polyacrylamide gel electrophoresis (PAGE) gel, and protein samples were transferred onto polyvinylidene fluoride (PVDF) membranes (Merck Millipore, USA, ISEQ00010). PVDF membranes were blocked by 5% skim milk (BD, USA, 232100) and were incubated first with rabbit primary antibodies overnight at 4°C, followed by incubation with a secondary antibody (Solarbio, China, SE134, 1:5000 dilution for western blot) for another 1 h. GAPDH (CST, USA, 5174, 1:1000 dilution for western blot) served as a loading control. The primary antibodies used for western in this study were as follows: LC3B-II (CST, USA 2775, 1:1000 dilution), p62 (CST, USA, 39749, 1:1000 dilution), Caspase-3 (CST, USA, 9662, 1:1000 dilution), Nod1 (CST, USA, 3545S, 1:1000 dilution), RIP2 (Abcam, UK, ab8428, 1:1000 dilution), p-ERK1/2 (CST, USA, 4370S, 1:1000 dilution), ERK1/2 (CST, USA, 4695, 1:1000 dilution), FOXO4 (CST, USA, 9472, 1:1000 dilution), p-IKKA (Abcam, UK, ab38515, 1:1000 dilution), IKKA (Abcam, UK, ab32041, 1:1000 dilution), BCL-2 (Abcam, UK, ab32124, 1:1000 dilution), BNIP3 (Abcam, UK, ab109362, 1:1000 dilution).

### Mongolian Gerbil *H. pylori* Infection Model

Five Mongolian gerbils weighing 60–80 g were used to establish the *in vivo* *H. pylori* model and five *H. pylori*-negative gerbils were used as controls. All gerbils were obtained from the Capital Medical University and were fed at secondary biosafety laboratories at the Chinese Center for Disease Control and Prevention. Gerbils were housed in standard plastic cages in a room with a 12-h light/dark cycle and free access to food and water throughout all experiments. Gerbils 6–8 weeks of age were infected with *H. pylori* ATCC 43504 strain solution by oral gavage with 0.5 mL 2×10^9^ CFU/mL. Gerbils were fasted for 12 h prior to challenge, and oral gavage was performed 5 times at intervals of 48 h. Before animals were euthanized, the ^13^C urea breath test and PCR were performed to confirm that *H. pylori* had colonized the gerbils. Ninety weeks after infection, gerbils were euthanized and the stomach tissue samples and blood were collected.

The animal experiments were conducted in accordance with the Guidelines of the CMU Animal Experiments and Experimental Animals Management Committee under a protocol approved by the Animal Experiments and Experimental Animal Welfare Committee of CMU (Permit number: AEEI-2016-154).

### TUNEL Staining

TUNEL staining was performed using the TUNEL Bright Green Apoptosis Detection Assay kit (Vazyme, China, A112) to detect apoptotic cells in the gerbil’s stomach according to the manufacturer’s instructions. Briefly, paraffin sections were dewaxed, hydrated, and treated with proteinase K for 30 min and then incubated with a fluorescently labeled solution of dUTP and TdT enzyme for 80 min at 37°C. Positive controls were incubated with DNase I for 10 min at room temperature prior to the fluorescent labeling procedure, while negative controls were incubated with dUTP for 10 min. The nuclei were then counterstained with Hoechst and the samples were blocked by antifading mounting medium.

### Immunohistochemistry

Stomach sections were incubated with rabbit monoclonal anti-LC3 antibody (Abcam, UK, ab128025, 1:100 dilution for immunohistochemistry) overnight at 4°C followed by incubation with corresponding biotinylated secondary antibody. The cell nuclei were counterstained with hematoxylin, and the samples were dehydrated in a gradient series, vitrified with dimethylbenzene, and finally mounted with neutral balsam.

### ELISA Analysis

The levels of BCL-2 and BNIP3 in the gerbils’ sera were measured by ELISA using the Gerbil BCL-2 ELISA kit (Enzymatic Biotechnology, China) and Gerbil BNIP3 ELISA kit (Enzymatic Biotechnology, China) following the manufacturer’s instructions. A 47 μL volume of sample dilution solution and 3 μL sample were added to each sample well of the enzyme labeling plate. After mixing, 100 μL enzyme labeling reagent was added into each well and incubated at 37°C for 1 h. The plate was washed, and the color developer was added at 37°C in a dark environment. The reaction was terminated after 15 min. Finally, the absorbance of each well was measured at 450 nm wavelength in a microplate reader.

### Statistical Analysis

All statistical analyses were carried out using SPSS v19.0 software. Data are expressed as the mean ± standard deviation. Independent sample *t*-test and one-way ANOVA analysis were used to determine the significance of the differences between the results. The Mann-Whitney U test was used to analyze gene expression from TCGA databases. Differences were considered statistically significant when the p-value was <0.05.

## Results

### Optimum Concentration of *H. pylori* Lysate Co-cultured With Gastric Epithelial Cells

**Figure 1:**
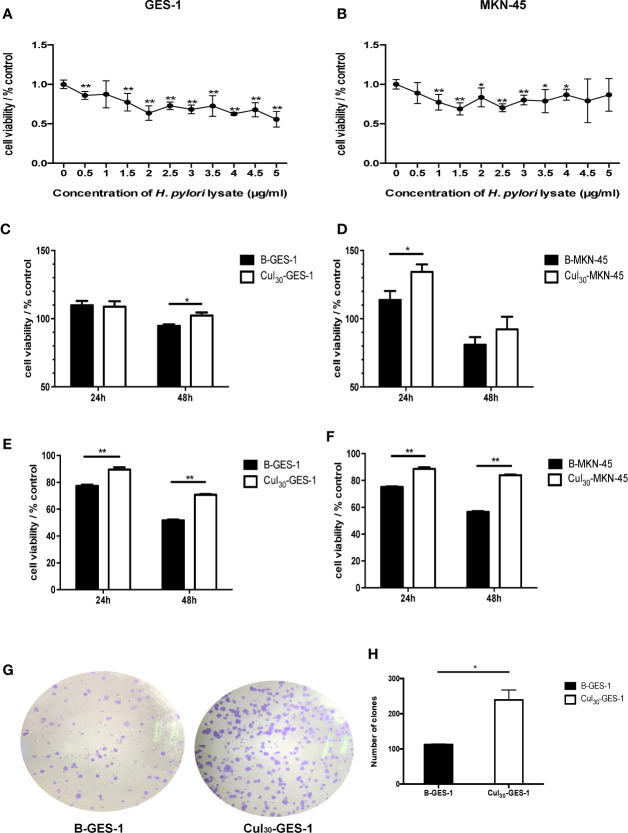
Sustained exposure to *H. pylori* lysate promotes proliferation of GES-1 and MKN-45 cells. GES-1 **(A)** and MKN-45 **(B)** cells were co-cultured with *H. pylori* lysate at a concentration of 0–5 μg/mL for 72 h, respectively. The concentration of *H. pylori* lysate exhibiting 70%–80% activity of normal cell activity was determined as the long-term co-culture concentration. Cul_30_-GES-1, B-GES-1, Cul_30_-MKN-45, and B-MKN-45 cells (n = 5 experiments) were challenged with *H. pylori* lysate (2 μg/mL for GES-1 cells and 1.5 μg/mL for MKN-45 cells) **(C, D)** or *H. pylori* (6×10^6^ CFU/mL) **(E, F)** for 24 h or 48 h, respectively. Cell viability was detected by the MTT assay. n = 5. **(G, H)** The proliferation of Cul_30_-GES-1 and B-GES-1 cells was detected by the plate cloning assay. Image J software was used to count the visible colonies (n = 3 experiments). *p < 0.05, **p < 0.01.

To select a suitable concentration of *H. pylori* lysate for long-term co-culture with cells, we tested different concentrations of lysate for co-culture with gastric epithelial cells for 72 h. The MTT assay showed that the cell activity decreased as the concentration of *H. pylori* lysate increased. To ensure the stable growth of cells in long-term co-culture with *H. pylori* lysate, the concentration of lysate with 70%–80% activity of normal cells was chosen as the optimum concentration, which was determined to be 2 μg/mL for GES-1 cells and 1.5 μg/mL for MKN-45 cells (**Figures 1A, B**). GES-1 cells and MKN-45 cells of the experimental group were cultured in medium with *H. pylori* lysate for 30 consecutive generations using the optimum concentration determined above, and they were labeled as Cul_30_-GES-1 and Cul_30_-MKN-45, respectively. The untreated normal cells were labeled as B-GES-1 and B-MKN-45.

### Sustained Exposure to *H. pylori* Lysate Promoted Proliferation of GES-1 and MKN-45 Cells

To study the regulation of *H. pylori* lysate on cell growth, Cul_30_-GES-1, B-GES-1, Cul_30_-MKN-45, and B-MKN-45 cells were cultured with *H. pylori* lysate (2 μg/mL for GES-1 cells and 1.5 μg/mL for MKN-45 cells) or *H. pylori* (6×10^6^ CFU/mL) (23) for 24 h or 48 h, respectively. The results of the MTT assay showed that the activities of Cul_30_-GES-1 and Cul_30_-MKN-45 cells treated with *H. pylori* lysate or *H. pylori* exceeded those of B-GES-1 and B-MKN-45 cells (**Figures 1C–F**), which was further verified by the colony formation assay of GES-1 cells (**Figures 1G, H**). These data indicated that long-term treatment of *H. pylori* lysate promoted the proliferation of GES-1 and MKN-45 cells. Meanwhile, the cells still maintained strong activity under long-term exposure to *H. pylori* lysate.

### Sustained Exposure to *H. pylori* Lysate Alters Migration and Invasion of Gastric Epithelial Cells

**Figure 2:**
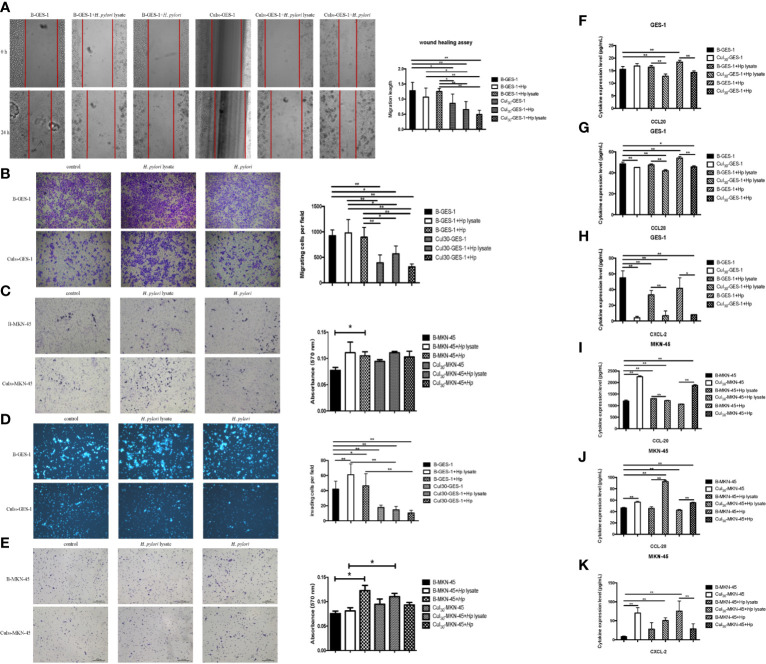
Sustained exposure to *H. pylori* lysate alters migration and invasion of GES-1 and MKN-45 cells. GES-1 or MKN-45 cells were treated with *H. pylori* lysate (2 μg/mL for GES-1 cells and 1.5 μg/mL for MKN-45 cells) or *H. pylori* (6×10^6^ CFU/mL) for 24 h. **(A)** The wound healing assay was used to determine the migration of Cul_30_-GES-1 and B-GES-1 cells exposure to *H. pylori* lysate or *H. pylori*. Red lines represent the borders of the wounds (n=5 experiments). **(B–E)** Transwell migration **(B, C)** and Transwell invasion **(D, E)** assays were performed to detect the migration and invasion ability of Cul_30_-GES-1, B-GES-1, Cul_30_-MKN-45, and B-MKN-45 cells exposed to *H. pylori* lysate or *H. pylori*. **(F–K)** Expression of CCL-20 **(F)**, CCL-28 **(G)**, and CXCL-2 **(H)** in GES-1 cells, and the expression of CCL-20 **(I)**, CCL-28 **(J)**, and CXCL-2 **(K)** in MKN-45 cells were measured by Luminex assays. n = 3. *p < 0.05, **p < 0.01.

To investigate whether *H. pylori* lysate would regulate the migratory and invasive ability of GES-1 and MKN-45 cells, Cul_30_-GES-1, B-GES-1, Cul_30_-MKN-45, and B-MKN-45 cells were cultured with *H. pylori* lysate (2 μg/mL for GES-1 cells and 1.5 μg/mL for MKN-45 cells) or *H. pylori* (6×10^6^ CFU/mL) for 24 h. The results of the wound healing assay showed that the migratory ability of Cul_30_-GES-1 exposed to *H. pylori* lysate or *H. pylori* was significantly inhibited compared with that of B-GES-1 cells (**Figure 2A**). The transwell migration assay yielded consistent results (**Figure 2B**). These data revealed that a 24-h exposure to *H. pylori* lysate had no significant effect on the migratory ability of GES-1 cells, but long-term exposure to *H. pylori* lysate could significantly inhibit GES-1 cell migration. Nevertheless, in MKN-45 cells, *H. pylori* lysate or *H. pylori* exposure only promoted the migratory ability of B-MKN-45 cells with no significant differences (**Figure 2C**), indicating that long-term exposure of *H. pylori* lysate did not inhibit migration of gastric cancer cells.

For the cell invasion assay, short-term exposure to *H. pylori* lysate or *H. pylori* promoted the invasive ability of B-GES-1 cells, but it inhibited the invasion of Cul_30_-GES-1 cells (**Figure 2D**). In addition, the invasive ability of Cul_30_-MKN-45 cells challenged by the *H. pylori* lysate increased significantly (**Figure 2E**). These results indicated that exposure to *H. pylori* lysate promoted the invasion of gastric epithelial cells but inhibited the invasion of cells co-cultured with *H. pylori* lysate. However, treatment of *H. pylori* lysate did not affect the invasive ability of gastric adenocarcinoma cells, but instead promoted the invasion of MKN-45 cells co-cultured with *H. pylori* lysate.

### Sustained Exposure to *H. pylori* Lysate Inhibited the Expression of CCL20, CCL28, and CXCL-2 of Gastric Epithelial Cells

The long-term exposure to *H. pylori* lysate inhibited the migration and invasion of normal gastric epithelial cells, but it had no effect on cancer cells. To investigate the mechanisms that altered migration and invasion of gastric cells, we evaluated the expression of cytokines CCL20, CCL28, and CXCL-2, which promote the migration and invasion of various cancer cells (24–26). Data showed that the expression of CCL20, CCL28, and CXCL-2 in Cul_30_-GES-1 cells under the treatment of *H. pylori* (6×10^6^ CFU/mL) or lysate (2 μg/mL for GES-1 cells and 1.5 μg/mL for MKN-45 cells) for 24 h significantly decreased relative to B-GES-1 cells (**Figures 2F–H**). However, the expression levels of these cytokines in Cul_30_-MKN-45 cells increased significantly compared with those of B-MKN-45 cells (p<0.01, **Figures 2I–K**). These results further confirmed that long-term exposure to *H. pylori* lysate altered migration and invasion of gastric epithelial cells.

### Autophagy of Gastric Epithelial Cells Was Inhibited by Persistent Treatment With *H. pylori* Lysate

**Figure 3:**
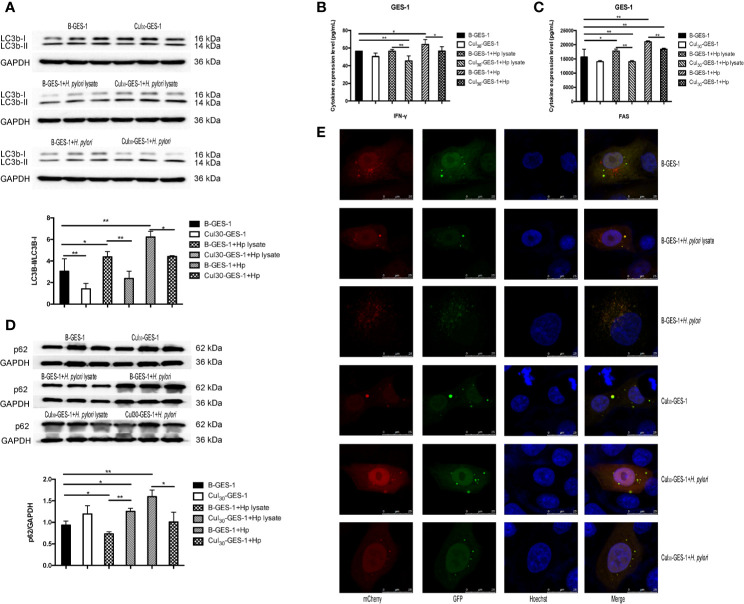
The treatment of *H. pylori* lysate inhibits autophagy of GES-1 cells. Cul_30_-GES-1 and B-GES-1 cells were challenged with *H. pylori* lysate (2 μg/mL) or *H. pylori* (6×10^6^ CFU/mL) for 24 h, and the expression of LC3b-II/LC3b-I **(A)** and p62 **(D)** in Cul_30_-GES-1 and B-GES-1 cells exposed to *H. pylori* lysate or *H. pylori* were detected by western blot. The expression of IFN-γ **(B)** and FAS **(C)** were measured by Luminex assays. **(E)** Representative fluorescence images of autophagosomes and autolysosomes in GES-1cells treated with *H. pylori* lysate using the tandem mCherry-EGFP-LC3 fusion protein assay. The autophagy flux was evaluated by the ratio of red spots to yellow spots. The yellow spots indicate autophagosomes, while the red spots indicate autolysosomes. If the phagosome and lysosome fuses normally, then the red fluorescence is greater than the yellow fluorescence. If downstream autophagy is blocked, the phagosome and lysosome cannot fuse normally, and then yellow fluorescence is the main color visualized. n=3. *p < 0.05, **p < 0.01.

After treating Cul_30_-GES-1 and B-GES-1 cells with *H. pylori* lysate (2 μg/mL) or *H. pylori* (6×10^6^ CFU/mL) for 24 h, the expression of an important indicator of autophagy LC3b-II (27) in B-GES-1 cells and Cul_30_-GES-1 cells treated with *H. pylori* lysate or *H. pylori* was upregulated in both cell lines. Further, the level of LC3b-II in *H. pylori*-treated B-GES-1 cells was significantly higher than that in cells exposed to lysate. Moreover, the expression of LC3b-II in Cul_30_-GES-1 cells co-cultured with *H. pylori* lysate or *H. pylori* was significantly lower than that in B-GES-1 cells (**Figure 3A**). These results demonstrated that long-term exposure of *H. pylori* lysate inhibited the autophagy of GES-1 cells. We then detected expression levels of vital autophagy regulator IFN-γ and FAS (28, 29) in B-GES-1 and Cul_30_-GES-1 cells under different conditions, and further confirmed the above results (**Figures 3B, C**).

The expression of LC3b-II and SQSTM1/p62 is regulated by the production and removal of autophagosomes (27). We next evaluated the expression of p62 in lysate-treated GES-1 cells. Western blot assays showed that the p62 level was inhibited in B-GES-1 cells after 24-h treatment of *H. pylori* lysate, while it was enhanced in Cul_30_-GES-1 cells (**Figure 3D**). Furthermore, we transfected B-GES-1 and Cul_30_-GES-1 cells with mCherry-EGFP-LC3 lentiviral vector. The EGFP signal in the mCherry-EGFP-LC3 fusion protein was quenched under acidic pH in autophagolysosomes, which allowed easier detection of autophagolysosomes (GFP-negative/RFP-positive; red dots) and autophagosomes (GFP positive/RFP positive; yellow dots) (**Figure 3E**). In B-GES-1 and lysate-treated B-GES-1 cells, the red puncta were more numerous than the yellow puncta, which was in contrast with Cul_30_-GES-1 cells (**Figure 3E**). These observations suggested that long-term treatment of *H. pylori* lysate might inhibit autophagy flux of GES-1 cells.

### Constant Treatment of *H. pylori* Lysate Inhibited Apoptosis of GES-1 Cells and Contributed to a Tendency for Cell Canceration

**Figure 4:**
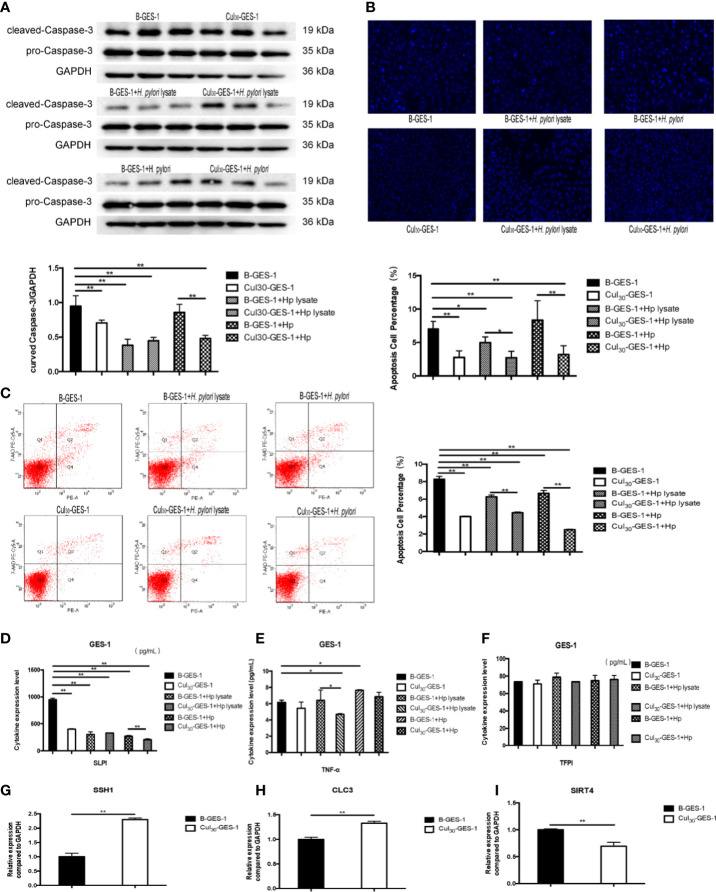
Continuous treatment of *H. pylori* lysate inhibits apoptosis of GES-1. Cul_30_-GES-1 and B-GES-1 cells were challenged with *H. pylori* lysate (2 μg/mL) or *H. pylori* (6×10^6^ CFU/mL) for 24 h and **(A)** the expression of cleaved Caspase-3 in Cul_30_-GES-1 and B-GES-1 cells exposed to *H. pylori* lysate or *H. pylori* were analyzed by Western blot. **(B)** Cells treated with *H. pylori* lysate or *H. pylori* stained with Hoechst 33342 (10 μg/mL) for 10 min. Nuclear morphologic changes were examined under a fluorescence microscope. **(C)** Cell apoptosis detected by flow cytometry. **(D–F)** The expression of SLPI **(D)**, TNF-α **(E)**, and TFPI **(F)** of B-GES-1 and Cul_30_-GES-1 cells were measured by Luminex assays. **(G–I)** Real-time qPCR results showing mRNA levels of *SSH1* **(G)**, *CLC3* **(H)**, and *SIRT4* **(I)** of Cul_30_-GES-1, and normalized to control cells. n=3. *p < 0.05, **p < 0.01.

We next identified whether *H. pylori* lysate would regulate the apoptosis of GES-1 cells. After being challenged by *H. pylori* lysate (2 μg/mL) or *H. pylori* (6×10^6^ CFU/mL) for 24 h, the expression of caspase-3 in Cul_30_-GES-1 cells decreased significantly, compared with that of B-GES-1 cells (**Figure 4A**). We also stained the nucleus of GES-1 cells with Hoechst and performed flow cytometry analysis, and the results further supported the above results (**Figures 4B, C**). In addition, we evaluated the expression of TNF-α, SLPI, and TFPI, which promote cell death (30–32). The expression levels of SLPI in Cul_30_-GES-1 cells treated with *H. pylori* or *H. pylori* lysate were significantly decreased compared to the control group (**Figure 4D**). After continuous co-culture with *H. pylori* lysate, the expression of TNF-α in Cul_30_-GES-1 cells showed a decreased trend (**Figure 4E**). However, there were no significant differences in the expression of TFPI between the two groups (**Figure 4F**).

Given that persistent stimulation by *H. pylori* lysate exposure promoted proliferation and inhibited autophagy and apoptosis of GES-1 cells, we hypothesized that the treatment could promote the tendency for malignant progression. We detected the mRNA expression level of three gastric cancer biomarkers, chloride channel-3 (*CLC-3*), slingshot protein phosphatase 1 (*SSH1*), and sirtuin 4 (*SIRT4*) (33–35) in Cul_30_-GES-1 and B-GES-1 cells. We found that the mRNA levels of *SSH1* and *CLC3* increased significantly, while SIRT4 decreased, compared with the control group (**Figures 4G–I**). These data suggested that long-term stimulation by *H. pylori* lysate may contribute to the tendency of malignant transformation of gastric epithelial cells.

### Screening of Genes Involved in *H. pylori*-Induced Gastric Cancer Based on TCGA Database

**Figure 5:**
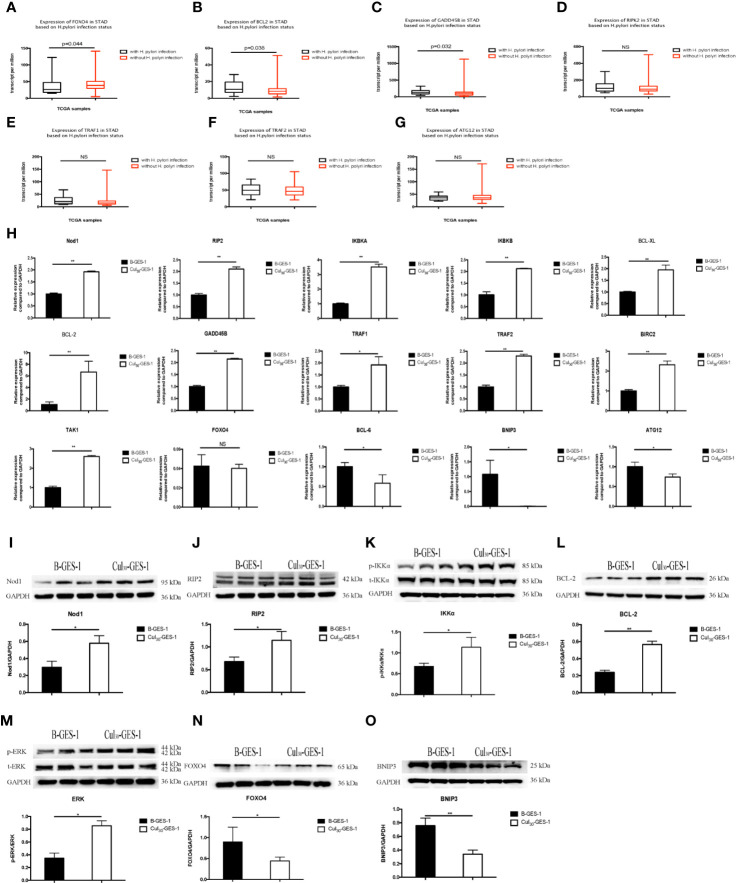
Pathways related to the inhibition of autophagy and apoptosis of gastric epithelial cells sustained exposure to *H. pylori* lysate. **(A–F)** Gene expression among *H. pylori*-positive (n=17) and *H. pylori*-negative (n=115) patients with gastric adenocarcinoma analyzed from the TCGA database. Down-regulation of *FOXO4* **(A)** and *ATG12* **(G)** is shown in *H. pylori*-positive samples, while *BCL2* **(B)**, *GADD45B* **(C)**, *RIP2* **(D)**, *TRAF1* **(E)**, and *TRAF2* **(F)** are upregulated. **(H)** mRNA expression of related genes in B-GES-1 and Cul_30_-GES-1 cells tested by Real-time qPCR. **(I–O)** Expression of Nod1, RIP2, p-IKKA/IKKA, BCL-2, p-ERK/ERK, FOXO4, and BNIP3 measured by Western blot. n=3. *p < 0.05, **p < 0.01. NS, No Significance.

To determine potential pathways affected by persistence stimulation by *H. pylori* lysate, we downloaded a dataset from a patient cohort with gastric adenocarcinoma from the TCGA database (https://cancergenome.nih.gov/). We divided the data into two groups according to patients with (n=17) or without *H. pylori* infection (n=115). We found that compared with *H. pylori* negative group, *H. pylori* infection induced the downregulation of the forkhead box O4 (*FOXO4*) gene (**Figure 5A**), upregulation of B-cell lymphoma-2 (*BCL2*), and upregulation of growth arrest and DNA-damage-inducible Beta (*GADD45B*) genes (**Figures 5B, C**). Moreover, *H. pylori* infection resulted in upregulation of *RIPK2*, TNF receptor-associated factor (*TRAF*)1 and *TRAF2* genes, and downregulation of autophagy related 12 homolog (*ATG12*) genes, although these differences were not statistically significant (**Figures 5D–G**).

### The Nod Receptor Pathway Was Related to the Inhibition of Autophagy and Apoptosis of Gastric Epithelial Cells Under Persistent Treatment of *H. pylori* Lysate

Previous studies have indicated that *Nod1-RIP2* regulates apoptosis through the NF-κB pathway and also regulates autophagy (36). Therefore, we evaluated the mRNA expression levels of *Nod1*, receptor-interacting protein 2 (*RIP2*), IκB kinase-α (*IKBKA*), and IκB kinase-β (*IKBKB*). All genes were significantly upregulated in Cul_30_-GES-1 cells, compared with B-GES-1 cells (**Figure 5H**). We then explored the mRNA levels of apoptosis-related downstream genes. B-cell lymphoma-XL (*BCL-XL*), *BCL-2*, *GADD45B*, *TRAF1*, *TRAF2*, and baculoviral IAP repeat-containing protein 2 (*BIRC2*) were upregulated in Cul_30_-GES-1 cells (**Figure 5H**). Moreover, we verified the protein levels of these genes. In accordance with the results above, the protein levels of Nod1, RIP2, IKKα, and BCL-2 increased significantly (**Figures 5I–L**). These results suggested that long-term exposure to *H. pylori* lysate may regulate the apoptosis of gastric epithelial cells through the *Nod1-RIP2-NF-κB* pathway.

*Nod1/RIP2* inhibits *FOXO4* expression through the mitogen-activated protein kinase *(MAPK)/ERK* pathway (37). Thus, we verified the mRNA and protein levels of related pathway and vital genes. The results indicated that the mRNA level of *TAK1* was significantly higher than that of the control group, while the mRNA level of *FOXO4* was inhibited, although there were no statistically significant differences (**Figure 5H**). The mRNA levels of B-cell lymphoma-6 (*BCL-6*), *BNIP3*, and *ATG12* were also downregulated (**Figure 5H**). Western blot showed that the phosphorylation level of extracellular regulated protein kinases (ERK) increased (**Figure 5M**), while the expression of FOXO4 and BNIP3 decreased significantly (**Figures 5N, O**). These results suggest that long-term exposure of *H. pylori* lysate may regulate apoptosis and autophagy of gastric epithelial cells through the *Nod1-RIP2-MAPK/ERK-FOXO4* pathway.

### Long-Term Infection of *H. pylori* Inhibited Autophagy and Apoptosis of Gastric Epithelial Cells *In vivo*

**Figure 6:**
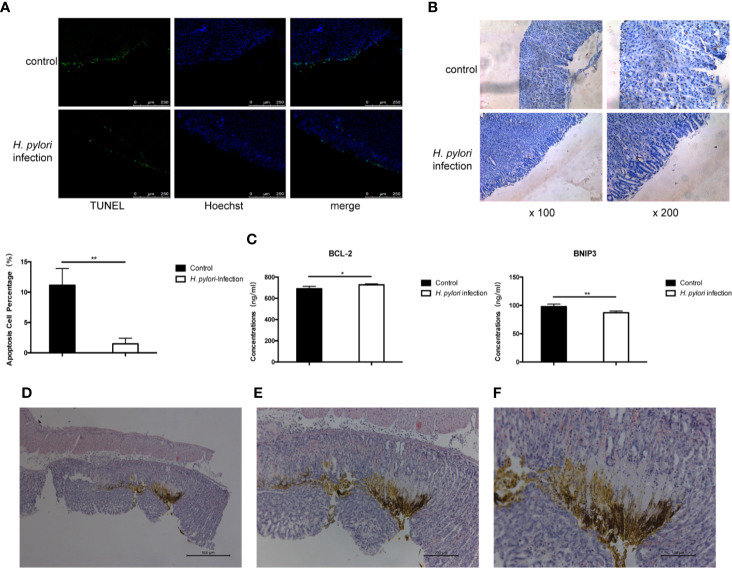
Long-term infection of *H. pylori* inhibits autophagy and apoptosis of gastric epithelial cells *in vivo*. Mongolian gerbils were infected by *H. pylori* for 90 weeks. Serum and gastric tissue were collected. **(A)** TUNEL staining assay and immunohistochemistry **(B)** of LC3 was performed to determine the apoptosis and autophagy of gerbil gastric epithelial cells. **(C)** The serum levels of BCL-2 and BNIP3 in gerbils (tested by ELISA; n=5). *p < 0.05, **p < 0.01. **(D–F)** HE staining revealing the pathology of Mongolian gerbil stomach samples. Representative histologic images from *H. pylori-*infected gerbils at original magnification ×50 **(D)**, ×100 **(E)**, and ×200 **(F)**.

We presumed that *H. pylori* proliferates continuously in the course of chronic infection. *H. pylori* and its lysate are in constant contact with gastric epithelial cells *in vivo*. Autophagy and apoptosis of cells may be inhibited, which is conducive to the sustained colonization of *H. pylori*, and this may promote a tendency toward progression to gastric cancer. We then carried out *in vivo* experiments to verify the results of the above *in vitro* results. Mongolian gerbils were infected with the *H. pylori* 43504 strain for 90 weeks and then the gastric tissues were collected. The TUNEL staining assay and immunohistochemistry were performed to determine the degree of apoptosis and autophagy induced in gastric epithelial cells. Compared to the control group, the apoptosis and autophagy of gerbils continuously exposed to *H. pylori* infection were remarkably inhibited (**Figures 6A, B**). Bcl-2 and BNIP3 are two key regulators of autophagy and apoptosis (38). The expression of BNIP3 has also been reported to be absent in gastric cancer (39). The serum levels of Bcl-2 and BNIP3 in gerbils were also tested, and the results were consistent with the data above (**Figure 6C**). Furthermore, hyperplastic lesions were identified in the stomach tissue of gerbils infected with *H. pylori* (**Figures 6D, F**), showing a tendency of gastric lesions to transform to cancer.

## Discussion

The global infection rate of *H. pylori* is about 44% (40). *H. pylori* is the most important risk factor for the development of gastric cancer (41). In the process of colonization, large amounts of *H. pylori* bacteria die and breakdown naturally, releasing lysate components, which influence the host cell in several ways. For example, its OMVs bind with pattern recognition receptors (PRR) on the surface of the host cell and regulate important cytological functions, including migration, invasion, apoptosis, autophagy, and carcinogenesis (22, 42, 43). However, there are some limitations that may have influenced the results of these studies. One is that the exposure time to bacterial lysate is generally too short to simulate an effective *H. pylori* infection *in vivo*. In addition, notably, when the components of the *H. pylori* lysate were isolated, they were antagonistic to each other in regulating some of the functions of the host cells. For instance, VacA induced apoptosis, while CagA blocked this effect and inhibited apoptosis. On the contrary, VacA also inhibited cytoskeleton deformity induced by CagA (27, 28). A single bacterial toxin cannot completely replace the regulatory effects of *H. pylori* on host cells. Therefore, it is necessary to use a *H. pylori* lysate instead of live bacteria to study the regulation of the host cell function by the continuous exposure to *H. pylori* infection. Considering that gastric epithelial cells are known to be initial contact points of bacteria in the gastric mucosa during *H. pylori* infection (44), human gastric epithelial cells GES-1 were used in our study. We co-cultured *H. pylori* lysate with GES-1 and MKN-45 cells for 30 generations and established a cell model of chronic stimulation of *H. pylori* lysate able to simulate the long-term symbiosis of *H. pylori* and host cells more closely, and to explore any relevant pathogenesis.

Cell proliferation is related to apoptosis, autophagy, and carcinogenesis. However, the effects of *H. pylori* and *H. pylori* lysate on the proliferation of gastric epithelial cells are not consistent. *In vitro* experiments showed that *H. pylori* promoted proliferation of gastric epithelial cells at low concentrations but promoted apoptosis at higher concentrations (45). Nonetheless, the effects of *H. pylori* lysate were consistent with those of *H. pylori* (46). It is generally believed that the contrasting results are due to the various types of *H. pylori* strains used and the different gastric epithelial cells with which they interacted. In this study, we found that the lysate of the *H. pylori* ATCC 43504 strain inhibited proliferation of B-GES-1 and B-MKN-45 cells dose-dependently. To ensure the survival of cells and achieve the goal of long-term co-culture of cells and *H. pylori* lysate, we chose a concentration of *H. pylori* lysate when the cell value-added rate was about 70%–80% as the optimum concentration. After continuous co-culture with *H. pylori* lysate, proliferation of Cul_30_-GES-1 cells significantly increased, which may be due to the tolerance of cells to *H. pylori* lysate.

Cell invasion and migration are two important characteristics in the process of cell carcinogenesis. *H. pylori* infection promotes migration and invasion of gastric epithelial cells in a CagA-dependent manner (47). CagA is translocated into the host cell mainly by the T4SS of *H. pylori* (48) and interacts with E-cadherin (49), resulting in the increase of movement and elongation of the host cell (50). In this study, differently from previous studies, we found that constant exposure to *H. pylori* lysate inhibited the migration and invasion of GES-1 cells. This may be due to the brief *H. pylori* stimulation, only 24 h, while we challenged gastric cells for 30 generations. Furthermore, ultrasonic lysis was used to prepare *H. pylori* lysate, and this process destroyed the T4SS, allowing only a small amount of CagA to enter the host cells through OMVs, and thus its promoting effect on cell migration and invasion may have been antagonized by other components, like lipopolysaccharide (LPS) (43). It is worth noting that differently from the effects on GES-1 cells, constant stimulation by *H. pylori* lysate showed less inhibition on the migration and invasion potential of MKN-45 cells, which may be due to the malignant properties of the cells. Although originating from normal cells, cancer cells have unique biological characteristics and behavior. For example, studies found that the mRNA and protein levels of HER2 in MKN-45 cells are significantly higher than that in GES-1 cells, and HER2 is subsequently proved to promote the migration and invasion of gastric cancer cells by upregulating CXCR4 (51). The higher migration and invasion characteristics of cancer cells may explain the reduced inhibitory effects of *H. pylori* lysate on the migration and invasion of MKN-45 cells.

Autophagy disorders interfere with health and disease (52). Many studies have shown that the inflammatory pathway promotes tumor progression by regulating autophagy (53). When *H. pylori* infects gastric epithelial cells for a short time, VacA and urease induce autophagy (54), but after a prolonged co-culture, *H. pylori* destroys the autophagy pathway and accumulates cells due to dysfunctional autophagy (7). We found that sustained exposure to *H. pylori* lysate blocked the autophagy flux of Cul_30_-GES-1 cells, resulting in a failure of fusion of autophagosomes and autolysosomes, and the accumulation of cells with defective autophagy. *H. pylori* can invade gastric epithelial cells and are isolated by lysosomal acidified autophagy (55) to promote survival and colonization (56, 57). Our results suggest that *H. pylori* lysates play vital roles in the process of chronic infection and inhibit the autophagy of host cells, which contributes to the survival and colonization of *H. pylori*.

The dysregulation of autophagy and apoptosis has adverse effects on the body and may even lead to cancer. It is believed that autophagy induced by *H. pylori* is associated with apoptosis, while autophagy occurs earlier than apoptosis (58). It has been reported that *H. pylori* infection inhibits apoptosis of the gastric epithelial cell (59, 60). We found that long-term stimulation of *H. pylori* lysate also inhibited the apoptosis of gastric epithelial cells, and we assumed that long-term exposure to *H. pylori* lysate may contribute to the initiation of the malignant transformation of the cell. To further evaluate the potential carcinogenic effects of exposure to *H. pylori* lysate, we detected mRNA levels of *CLC-3*, *SSH1*, and *SIRT4*, and the results confirmed our hypothesis. Sustained exposure to *H. pylori* lysate promoted the proliferation of gastric epithelial cells, inhibited autophagy and apoptosis, and facilitated the survival and colonization of bacteria, which may further promote the malignant transformation of cells. This was also confirmed by results *in vivo*. We infected Mongolian gerbils with *H. pylori* 43504 strain for 90 weeks. Although no carcinogenesis was evidenced in the stomach, dysplasia was present. Gastric epithelial dysplasia is a crucial pathology stage of the Correa cascade leading to gastric cancer (61). The observed dysplasia in model indicates that the pathological changes in the stomach of infected gerbils were indicative of transformation into cancer. These data suggest that *H. pylori* lysate acts as an “accomplice” in the process of *H. pylori-*induced gastric diseases.

Subsequently, we explored the underlying pathways involved in the long-term exposure cell model. Through the screening of clinical data and cell experiments, we found that continuous stimulation of *H. pylori* lysate upregulated mRNA and protein levels of *Nod1-RIP2-NF-κB* and of downstream genes *BCL-2* and *GADD45B*, which is consistent with previous reports. The *NF-κB* pathway regulates cell apoptosis (60) and the regulation of *BCL-2* by *NF-κB* plays an important role in host cell apoptosis induced by *H. pylori* infection (62). *H. pylori* activates *NF-κB*, inflammation and gastric cancer *via Nod1*-dependent activation (63). Furthermore, Nod1 is a member of the Nod-like receptor (NLR) family, a cytoplasmic recognition receptor in cells, which recognizes a variety of ligands, including peptidoglycan (PGN) and flagellin from bacterial pathogens and viral and bacterial RNA (19). The *Nod1* mRNA expression level was also shown to be upregulated in gastric cancer tissues (64). These results suggested that *H. pylori* lysate may regulate apoptosis of gastric epithelial cells *via* the *Nod1-RIP2- NF-κB* pathway. In addition, we found that long-term stimulation of *H. pylori* lysate promoted the phosphorylation of *ERK*, and then inhibited the levels of *FOXO4* and its downstream genes, *BCL-6*, *BNIP3*, and *ATG12*, which is consistent with reports indicating that the *FOXO* pathway regulates cell autophagy and apoptosis (65, 66).

**Figure 7:**
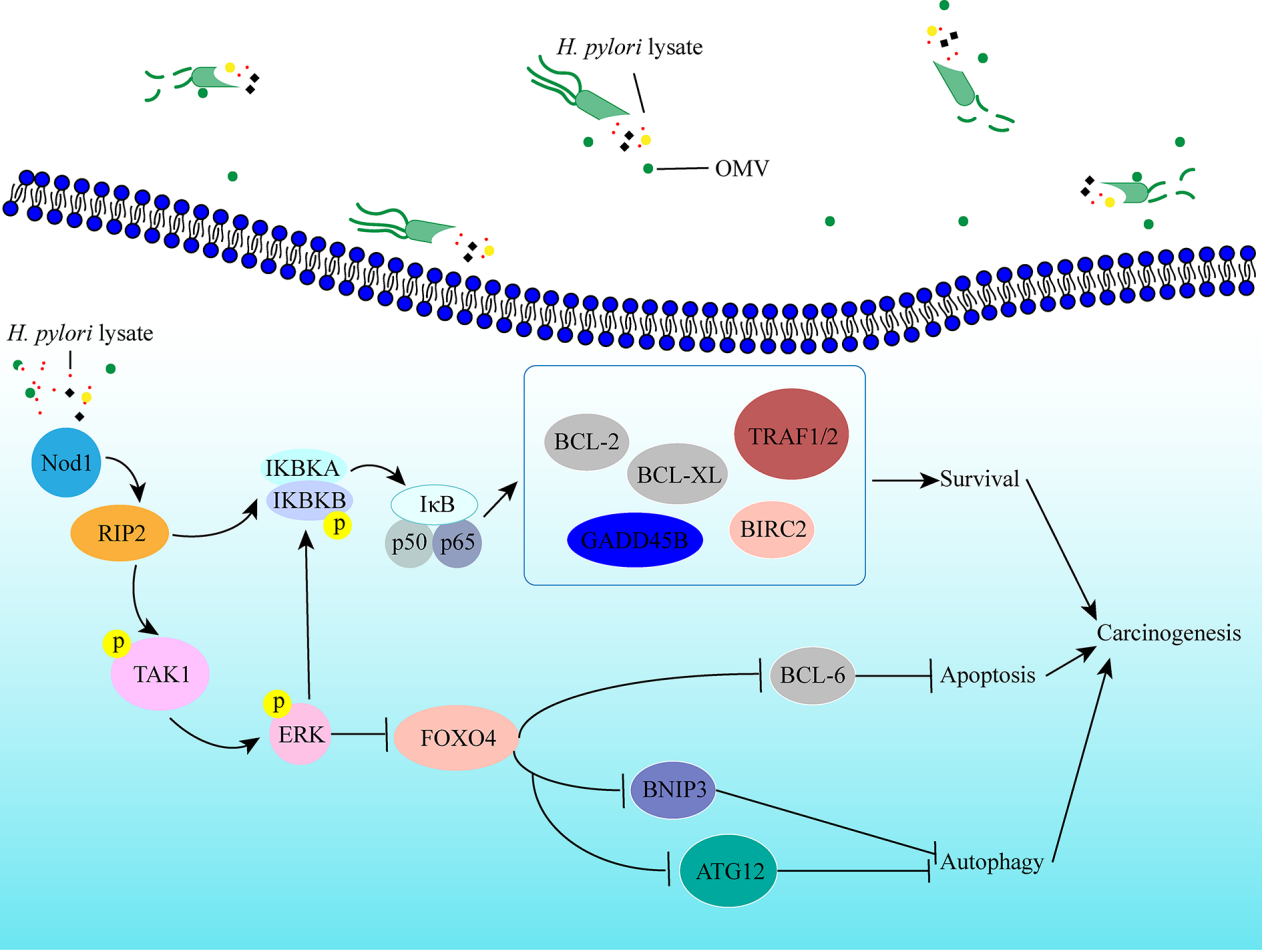
Schematic representation of the signaling pathways induced by prolonged exposure to *H. pylori*. Sustained exposure to *H. pylori* lysate inhibits apoptosis and autophagy of gastric epithelial cells *via* the *Nod1-NF-κB/MAPK-ERK/FOXO4* pathway, which promotes cells survival, and may contribute to the tendency toward cell malignant transformation.

In conclusion, we established a long-term gastric epithelial cell line model co-culture with *H. pylori* lysate to explore the effects of sustained exposure to *H. pylori* lysate on gastric cells, and we found that continuous treatment of *H. pylori* lysate promoted gastric epithelial cell proliferation and inhibited cell autophagy and apoptosis *via* the *Nod1-NF-κB/MAPK-ERK/FOXO4* pathway (**Figure 7**). In the process of *H. pylori-*induced gastric lesions, *H. pylori* lysate acts as an “accomplice.”

## Data Availability Statement

Publicly available datasets were analyzed in this study. This data can be found here: https://www.cancer.gov/about-nci/organization/ccg/research/structural-genomics/tcga.

## Ethics Statement

The animal study was reviewed and approved by the Animal Experiments and Experimental Animal Welfare Committee of CMU (Permit number: AEEI-2016-154), Capital Medical University.

## Author Contributions

All authors contributed to the study conception and design. ZC and CL designed the study. YH and CW conducted the experiments. YH created the figures and wrote the manuscript. XZ conducted the animal experiments. XLu mainly provided technical and material support. JX cultivated *H. pylori* and prepared *H. pylori* lysate. JLv evaluated the gene expression *via* the TCGA database. MG, XH, XLi, JLu, and XD did analysis and interpretation of data. All authors contributed to the article and approved the submitted version.

## Funding

This work was supported by the National Natural Science Foundation of China (No. 32070537, 31772545, 31970512, 31872308, 83902332), High-level Teachers in Beijing Municipal Universities in the Period of 13th Five Plan (No. IDHT20170516), National Key Research and Development Plan of China (No. 2017YFD0501602), and Beijing Science and Technology Program (D181100000518002).

## Conflict of Interest

The authors declare that the research was conducted in the absence of any commercial or financial relationships that could be construed as a potential conflict of interest.

## Abbreviations

*H. pylori*, *Helicobacter pylori;* T4SS, type IV secretion system; CagA, cytotoxin associated gene product A; VacA, vacuolating cytotoxin A; OMV, outer membrane vesicles; LPS, lipopolysaccharide; PGN, peptidoglycan; Ure, urease; STAT3, signal transducer and activator of transcription 3; ATCC, American type culture collection; PBS, phosphate buffer saline; BCA, bicinchoninic acid; DMEM, dulbecco’s modified eagle medium; FBS, fetal bovine serum; MTT, 3-(4, 5-dimethyl-2-thiazolyl)-2,5-diphenyl-2-H-tetrazolium bromide; DMSO, dimethyl sulfoxide; EGFP, enhanced green fluorescent protein; 7-AAD, 7-Aminoactinomycin D; TCGA database, The Cancer Genome Atlas database; GAPDH, glyceraldehyde 3-phosphate dehydrogenase; RIP2, receptor-interacting protein 2; MAPK, mitogen-activated protein kinase; NF-κB, nuclear factor kappa B; ERK, extracellular regulated protein kinases; p-ERK, phosphorylated extracellular regulated protein kinase; FOXO4, forkhead box O4; IKKA/IKBKA, IκB kinase-α; p-IKKA, phosphorylated IκB kinase-α; BCL-2, B-cell lymphoma-2; BNIP3, BCL2 interacting protein 3; BCL-XL, B-cell lymphoma-XL; GADD45B, growth arrest and DNA-damage-inducible Beta; TRAF1/2, TNF receptor-associated factor ½; BIRC2, baculoviral IAP repeat-containing protein 2; ATG12, autophagy related 12 homolog; dUTP, 2’-deoxyuridine 5’-triphosphate; TdT enzyme, terminal deoxynucleotidyl transferase; PRR, pattern recognition receptor; NLR, Nod like receptor; CCL20, C-C motif chemokine ligand 20; CCL28, C-C motif chemokine ligand 28; CXCL-2, C-X-C motif chemokine ligand 2; IFN-γ, interferon- γ; FAS, cell surface death receptor; TNF-α, tumor necrosis factor- α; SLPI, secretory leukocyte peptidase inhibitor; TFPI, tissue factor pathway inhibitor; CLC-3, chloride channel-3; SSH1, slingshot protein phosphatase 1; SIRT4, sirtuin 4.
