# Supplementary material for: Sustained Exposure to Helicobacter pylori Lysate Inhibits Apoptosis and Autophagy of Gastric Epithelial Cells
Source: Front Oncol. 2020 Oct 29;10:581364. doi: 10.3389/fonc.2020.581364 (PMC7658535; doi:10.3389/fonc.2020.581364)
Supplement: Supplementary file 1 [file Table_1.docx]

**Supplementary Table 1**

| Gene | Forward | Reverse |
| --- | --- | --- |
| Nod1 | 5'-TGGTGGCCAAGTGATTGTAAGT-3' | 5'-TCTGTAATCGCCGCCACAAT-3' |
| RIP2 | 5'-CGCTGCTCGACAGTGAAAGA-3' | 5'-TTCAGGCTCATTGCAAATTCCC-3' |
| IKBKA | 5'-GTGCAGTAACCCCTCAGACA-3' | 5'-TTTGTGCTGAAGTCTCCCACA-3' |
| IKBKB | 5'-GACAGTGGGAAATAACACCCGA-3' | 5'-AGCCCTTAGCCCAACATCCT-3' |
| BCL-XL | 5'-GATGGGGTAAACTGGGGTCG-3' | 5'-AGGTAAGTGGCCATCCAAGC-3' |
| BCL-2 | 5'-GGTGGGGTCATGTGTGTGG-3' | 5'-CGGTTCAGGTACTCAGTCATCC-3' |
| GADD45B | 5'-GCCCTGCAAATCCACTTCAC-3' | 5'-GTGTGAGGGTTCGTGACCAG-3' |
| TRAF1 | 5'-TAGGCGGTGGCGGAGG-3' | 5'-ATCCCCTGGATGGTGACTGA-3' |
| TRAF2 | 5'-CTCGGTGTGAGCAAGTGGAC-3' | 5'-GCTAGCTGCAGCCATGAGAG-3' |
| BIRC2 | 5'-GGCCGTATCTCCTTGTCGG-3' | 5'-TGCAGGGGGACAAAATAGGG-3' |
| TAK1 | 5'-CCAACCTCTGAGGGCAAGAG-3' | 5'-CTGTCCGTTGCCTGTGGTT-3' |
| FOXO4 | 5'-CCAGAGATCGCTAACCAGCC-3' | 5'-CTTTCAATGGCCTTTTCCCCC-3' |
| BCL-6 | 5'-TGTTTGAGGATCCCTTCCATGA-3' | 5'-GAAGACACATGGGAGTGGGAG-3' |
| BNIP3 | 5'-GGTCAAGTCGGCCGGAAAA-3' | 5'-CAAAAGGTGCTGGTGGAGGTT-3' |
| ATG12 | 5'-AGTCTGTGTTGCAGCTTCCT-3' | 5'-GTCTGGGGAAGGAGCAAAGG-3' |
| SSH1 | 5'-AATCTTCAGCACCCCCACAA-3' | 5'-TACACCACCACCATGTACCG-3' |
| CLC3 | 5'-TCTGAGCAGCTGTTCCATAGAG-3' | 5'-GCTGCCTCCATTTGTCATTGT-3' |
| SIRT4 | 5'-GAGGAGTCTTTTATGGGCGGT-3' | 5'-CACTACACCAGCCTCTTCCAG-3' |

**Supplementary Table 1**. Nucleotide sequences of primers used for RT-PCR reactions.
